# Passive sensing data predicts stress in university students: a supervised machine learning method for digital phenotyping

**DOI:** 10.3389/fpsyt.2024.1422027

**Published:** 2024-08-26

**Authors:** Artur Shvetcov, Joost Funke Kupper, Wu-Yi Zheng, Aimy Slade, Jin Han, Alexis Whitton, Michael Spoelma, Leonard Hoon, Kon Mouzakis, Rajesh Vasa, Sunil Gupta, Svetha Venkatesh, Jill Newby, Helen Christensen

**Affiliations:** ^1^ Black Dog Institute, University of New South Wales, Sydney, NSW, Australia; ^2^ Applied Artificial Intelligence Institute, Deakin University, Burwood, VIC, Australia; ^3^ Applied Artificial Intelligence Institute, Deakin University, Geelong, VIC, Australia

**Keywords:** university student, digital phenotype, stress, passive sensing, machine learning

## Abstract

**Introduction:**

University students are particularly susceptible to developing high levels of stress, which occur when environmental demands outweigh an individual’s ability to cope. The growing advent of mental health smartphone apps has led to a surge in use by university students seeking ways to help them cope with stress. Use of these apps has afforded researchers the unique ability to collect extensive amounts of passive sensing data including GPS and step detection. Despite this, little is known about the relationship between passive sensing data and stress. Further, there are no established methodologies or tools to predict stress from passive sensing data in this group.

**Methods:**

In this study, we establish a clear machine learning-based methodological pipeline for processing passive sensing data and extracting features that may be relevant in the context of mental health.

**Results:**

We then use this methodology to determine the relationship between passive sensing data and stress in university students.

**Discussion:**

In doing so, we offer the first proof-of-principle data for the utility of our methodological pipeline and highlight that passive sensing data can indeed digitally phenotype stress in university students.

**Clinical trial registration:**

Australia New Zealand Clinical Trials Registry (ANZCTR), identifier ACTRN12621001223820.

## 1 Introduction

Stress is the physiological and psychological response when environmental demands outweigh an individual’s ability to cope (1). Although brief exposures to low levels of stress are normal and even possibly beneficial for performance (2), chronic stress can lead to serious consequences including depression, burnout, and disease (1, 3–5). Early adulthood is characterized by rapid changes, both physiological and psychological. Further, there are many new challenges during this time including changing environments (e.g. moving to a new city), deadline pressures, increased social interaction and balancing school and work demands. Combined, these changes and pressures make university students particularly vulnerable to stress (6–8). They are well-documented to develop signs of stress-induced psychological distress, which can lead to low academic achievement, interpersonal problems, depression, burnout, self-harm, and suicide (9, 10).

Numerous mental health self-help mobile applications have been developed to combat university students’ poor mental health (11). Here, students who are feeling distressed can rapidly access affordable, or free, applications that aim to provide them with coping skills and strategies for self-help without the need for traditional face-to-face psychological sessions with a therapist. Further, mobile phones are increasingly accessible and popular for younger people and therefore present an easy tool by which to deliver digital health services (12). In line with this, younger adults show substantial interest in trying smartphone mental health apps, a phenomenon that was further increased by the recent COVID-19 pandemic (13–15). Students, in particular, are attracted to these types of apps due to immediate availability, convenience, confidentiality, and an ability to avoid the stigma associated with seeking face-to-face appointments with mental health specialists (12, 16). Additionally, students experiencing academic stress and burdens associated with transitioning to post-secondary institutions are known to seek out smartphone apps to help them cope (17).

Mental health smartphone apps have the benefit of being able to collect copious amounts of data from single users. Importantly, smartphones have multiple passive sensors that enable tracking of various aspects of users’ day to day lives. This passive sensing data includes GPS, which determines the location of the phone, an accelerometer and gyroscope to measure the acceleration in space and patterns of physical movement, and a step detector to estimate the number of steps. There has been a growing interest in using this passive sensing data to identify variables that can predict mental health status and outcomes. Variables that have been extracted from passive sensing data have been shown to be associated with mental health and psychiatric disorders including depression (18–21), stress (22, 23), anxiety (21), sleep quality (23), dementia (24), bipolar disease (25) and schizophrenia (26).

Despite the growing interest in the relationship between passive sensing data and mental health, little is known about its relationship with stress in university students. Stress in and of itself is an important adaptive mechanism of survival that helps the body to mobilize resources to respond to threat. However, the chronic activation of the stress response system can lead to catastrophic physical and mental health outcomes. More specifically, chronic stress has been shown to lead to depression (27), and problems with cardiovascular (28) and immune (29) systems. Smartphone-based interventions and tools that efficiently diagnose stress early are urgently needed to prevent these substantial health burdens.

Although previous research has shown a relationship between passive sensing data and mental health outcomes, there are limitations. First, there has been limited description of the methodologies used to both extract and process this type of data. For example, GPS data often has differences in the accuracy of determining coordinates due to a high dependency on factors including unobstructed receivers and good reception. Another example is that irregular smartphone internet connections can result in a data loss. In fact, a recent review highlighted that passive sensing papers were characterized by a high level of variability in the quality of reporting, including methodologies, that limited interpretability and reproducibility (30). It is imperative, therefore, to begin to work toward establishing clear methodologies that can translate into reproducible research using passive sensing data. Second, although some studies report a clear relationship between the two, others have not. For example, some studies show only a low to modest relationship between passive sensing data and mental health, depression, and anxiety (21, 31). Another required the use of synthetic data to improve passive sensing model performance in predicting sleep quality and stress in university students (23).

In this study, we had two central aims: (1) to establish a clear methodological pipeline for processing passive sensing data and extracting features that may be relevant in the context of mental health and (2) to use this methodology to determine the relationship between patterns of university students’ mobility, as indicated by passive sensing data, and their stress levels. In doing so, we offer the first proof-of-principle data for our methodological pipeline and, using supervised machine learning models, demonstrate that passive sensing data can indeed digitally phenotype stress in university students.

## 2 Method

### 2.1 Study design and participants

Mental health app user data was collected from the Vibe Up study (32). Vibe Up is a data collection application built for Android and iOS that uses an artificial intelligence algorithm to deliver the most effective mental health interventions to university student users in Australia. Participants of this study also completed survey-based mental health and wellbeing assessments throughout. Passive data, including accelerometer, gyroscope, activity monitoring, distance, and step count was collected across all 30 days of the study. Prospective users of the application were able to opt out from the passive sensing collection aspect of the study, therefore limiting the likelihood that users included in the present study were influenced by concerns with app tracking. Eligibility for participation was defined by the following: ≥ 18 years of age, currently attending a tertiary institution in Australia, remaining in Australia throughout the study period, and completed screening surveys. Users also had to have a Kessler Psychological Distress Scale (K10) score of ≥20 (33) and Suicidal Ideation Attributes Scale (SIDAS) (34) <21, to ensure that although users weren’t likely to be “well” they didn’t have a high level of suicidal ideation. A total of 409 participants were included in the present study. Using 10-fold cross-validation, we confirmed that this sample size was sufficient for the analyses in our study. The deviation between folds were 1.7%, indicating robust performance of the models. The study was approved by the University of New South Wales Human Research Ethics Committee, approval no. HC200466.

### 2.2 Questionnaires

At screening, users were asked questions about their demographic information including age, sex at birth, sexual orientation, language spoken at home, international or domestic student status, previous mental health diagnosis, and whether they used online mental health services in the past 12 weeks. Once users started the Vibe Up app, they completed the Depression and Stress Scale (DASS) three times across the study (35). Here, responses are encoded using 4-item rating scale ranging from ‘Did not apply to me at all’ (0) to ‘Applied to me very much, or most of the time’ (3). As stress was our primary outcome of interest (or output variable), we only used responses to the stress subscale. The level of stress was determined by summing the item scores, multiplying it by two, and converting it to a z-score using reference values for the mean (11.19) and standard deviation (8.25) of the general population of young adults aged 20-29. Further, participants were discretized based on the z-score into no stress group (z-score < 0.5), mild to moderate (z-score 0.5 to 2.0) and severe to extremely severe (z-score > 2.0) as has been done previously (36).

### 2.3 Passive sensing data collection

Passive data collection is managed by the Conductor Software Development Kit (SDK), which collects data based on predefined schedules. For Vibe Up, this collection period was all day for 30 days (the duration of each trial). The sample rates of each stream are unique based on the data being collected. GPS location is only recorded whenever a user has significantly moved. Similarly, activity monitoring, distance, and step count are only recorded if a user is actively moving. Accelerometer data is continuously recorded at 50 Hz on iOS and 60 Hz on Android. Gyroscope is recorded at 50 Hz, but on iOS this can only be collected while the app is in the foreground.

### 2.4 Passive sensing data feature selection

**Figure 1:**
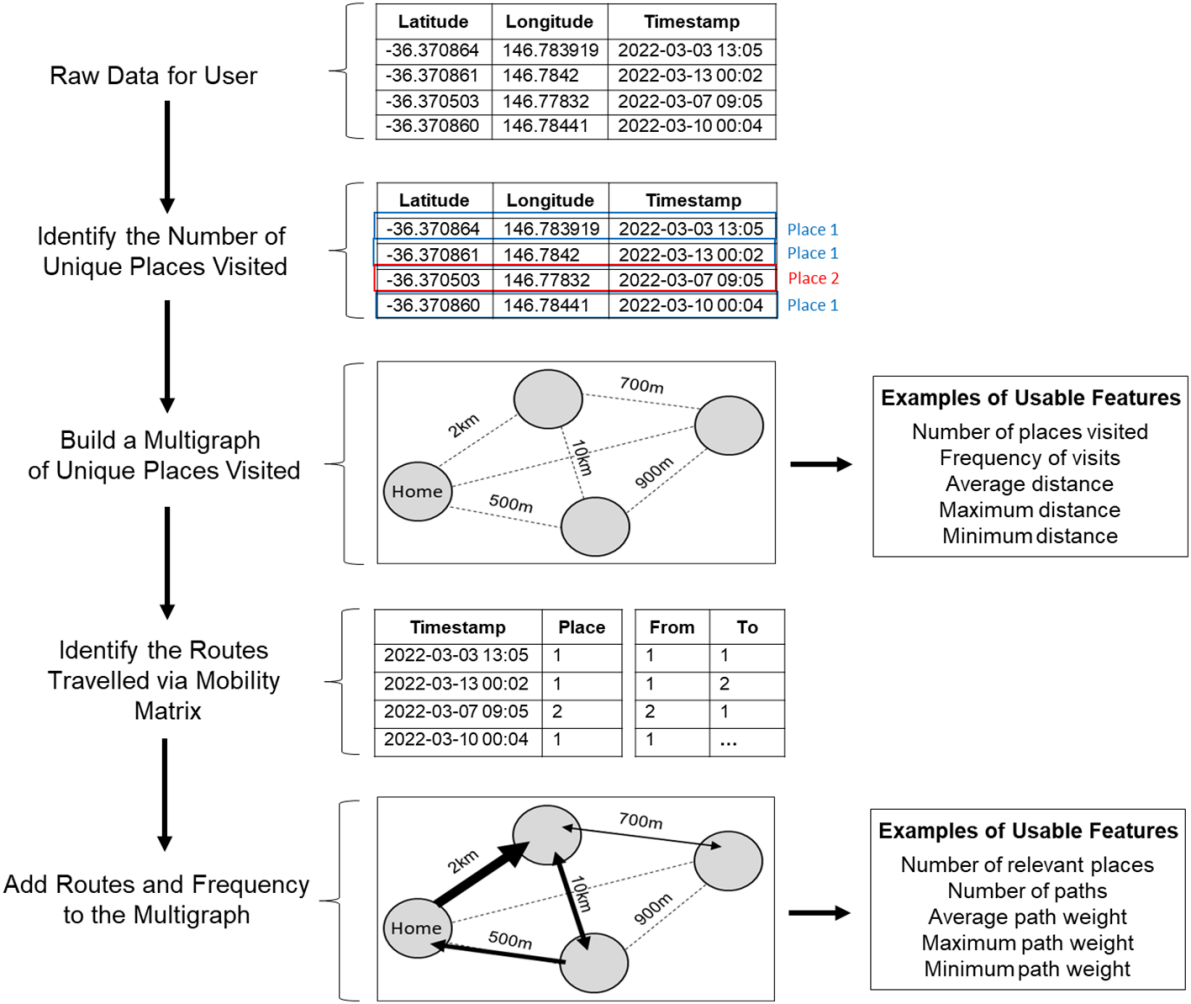
Schematic of the steps used to process raw passive sensing data collected from users’ mobile phones and identify usable features for subsequent statistical analyses and predictive modelling.

The use of passive sensing data is becoming more widespread in the literature on mobile app use. Despite this, there remains little consensus and few, if any, descriptions of the methods by which passive sensing data is processed and features are extracted for downstream statistical and predictive models. Here, we suggest an approach that focuses on building directed, edge-weighted graphs that capture the main features of user mobility patterns (**Figure 1**).

#### 2.4.1 Stage 1: processing raw passive sensing data

The first consideration is that GPS coordinates collected from mobile phone apps can have technical inaccuracies that cause a specific location to look different every time an estimation occurs (i.e. has slightly different GPS coordinates). Therefore, we present each location as a multigraph with area of 5000m^2^, considerably larger than the average property size in Australia, to reduce noise that may be caused by these inaccurate coordinates and randomly captured activities while someone is moving around their property. All coordinates that fall into this area are considered as one location. From this data, it is then possible to: (1) estimate the number of places a user visits, (2) calculate the distance between these places, (3) identify which location is likely to be home (highest number of occurrences/visits), and (4) identify which locations are likely to be irrelevant (lowest number of occurrences/visits). This data processed at this first stage can then be translated into usable features for statistical analyses and/or predictive models, including: number of places visited, frequency of visits, average distance, and maximum and minimum distances.

#### 2.4.2 Stage 2: characterize the routes and paths between places using mobility matrices

After identifying the number of unique places visited, we can then use the corresponding GPS timestamps to compute a mobility matrix for each user. The mobility matrix, therefore, contains information regarding date of visit, time of day (from 00:00 to 23:59), and the order of visited places at specific time intervals daily across the study. From here, we can then calculate routes and paths that a user has taken between places. Further, by using the number of passes between visited places (used to determine how often the route is used), computed in Stage 1, we can estimate the relevance of the routes between different places as well as the direction of travel. Importantly, unlike in Stage 1, Stage 2 data processing covers most aspects of human mobility: how many places a user visits, how many routes a user uses to arrive at those places and how often, the typical order of places visit, the time the working day starts and ends, average time a person spent at a particular place, what the night time lifestyle looks like (e.g. frequent night activities suggesting socialization), and so forth. Additionally, it is worth noting that some apps collect data about the types of movement, number of steps, and distance travelled. Once overlapped with the GPS dataset, it is possible to add this data to the geometric maps to determine the preferred way of travelling between places. Although we did attempt to collect this type of data in the present study, there was a lack of overlap between steps and GPS timestamps. Therefore, instead of merging these data together, we treated steps as a separate variable and summed up the number of steps per day. We then used quantiles (25%, 50%, and 75%), as well as maximum and minimum values as features. Overall, the data from Stage 2 can be extracted as several features including number of relevant places, number of paths, average path weight, and minimum and maximum path weights.

### 2.5 Analytical approach

To determine if the extracted passive sensing data features could predict stress, we deployed several machine learning algorithms including general linear algorithms [lasso and ridge regression, shrinkage discriminant analysis (SDA)], geometric distance-based algorithms [k-nearest neighbor (KNN)], tree-based algorithms (classification and regression trees (CART), random forest), and artificial neural networks (ANN). The dataset was split into a training dataset and testing, held-out dataset (70% and 30% respectively). Machine learning models were built, fine-tuned and validated on the training dataset by using three-fold cross-validation repeated five times. For the final assessment of machine learning models’ performance a held-out dataset was used. Where there were class imbalances of the output variable, an oversampling technique was used whereby the underrepresented class is randomly resampled to ensure that the algorithms receive approximately the same number of classes. For all algorithms, a fine-tuning grid method was used where all possible combinations of parameters within the predetermined ranges were estimated (**Table 1**).

**Table 1:** Parameters of the supervised machine learning algorithms used to predict stress based on passive sensing data features.

Model	Parameters
Linear regression	α: 0, 1 λ: 0.001-1
Shrinkage discriminant analysis (SDA)	Diagonal: true, false λ: 0.001-1
K-nearest neighbor (KNN)	Number of the nearest neighbors: 1-15
Classification and regression trees (CART)	Complexity parameter: 0.001-0.1
Random forest	Number of variables randomly sampled: 1-# of features in dataset
Artificial neural networks (ANN)	Number of hidden layers: 0 and 1 Number of neurons in hidden layers: 3-50 Activation function: tanh, relu, leaky relu Optimization algorithm: Adam, RMSprop, SGD Learning rate: 0.01-0.00001 Batch size: 16-32 Epochs: 10-100 Regularization dropout layer with probability: 0.1-0.8 Kernel regularizer: L1, L2

To estimate the performance of the binary classification models, we used area under the curve (AUC). This measure reflects the level of sensitivity and specificity of the model and thus general distinguishing capacity of the model. We also used precision, indicating the proportion of positive predictions is correct, recall, to indicate the proportion of positive cases that were predicted correctly and F1, which is a harmonic mean of precision and recall.

All inferential statistics were performed using Kruskal-Wallis followed by a *post-hoc* Dunn test for three samples comparisons. A Benjamini-Hochberg multiple correction was applied to adjust the *p* values and reduce the risk of a false positive. To determine the correlation between features and the output variable, a Pearson correlation coefficient was used. Analyses were performed in RStudio with R 3.6.3. Supervised machine learning was done using the caret package and neural networks were built using keras library.

## 3 Results

### 3.1 Participant characteristics

The demographic characteristics of the Australian university student users of the Vibe Up app are shown in **Table 2**. The average age of user was 23.6 (range 18 to 34). The majority of users identified as female (76%), spoke English at home (94%), and were domestic students (95%). Approximately half of the user group had a previous mental health diagnosis (54%) although comparatively fewer (25% of users) had used online mental health services in the past 12 weeks. A substantial number of users (39%) identified as being LGBTQIA+ (**Table 2**).

**Table 2:** Demographic characteristics of the university student users of the Vibe Up app.

Sample numbers	409
Age (average, ± SEM, and range)	23.6 ± 5.2 (18-34)
Sex at birth	76% female
Identify as LGBTQIA+	39%
Speak English at home	94%
Domestic student	95%
Previous mental health diagnosis	54%
Used online mental health service in the past 12 weeks	25%

### 3.2 Features show weak relationship with stress z-scores

**Figure 2:**
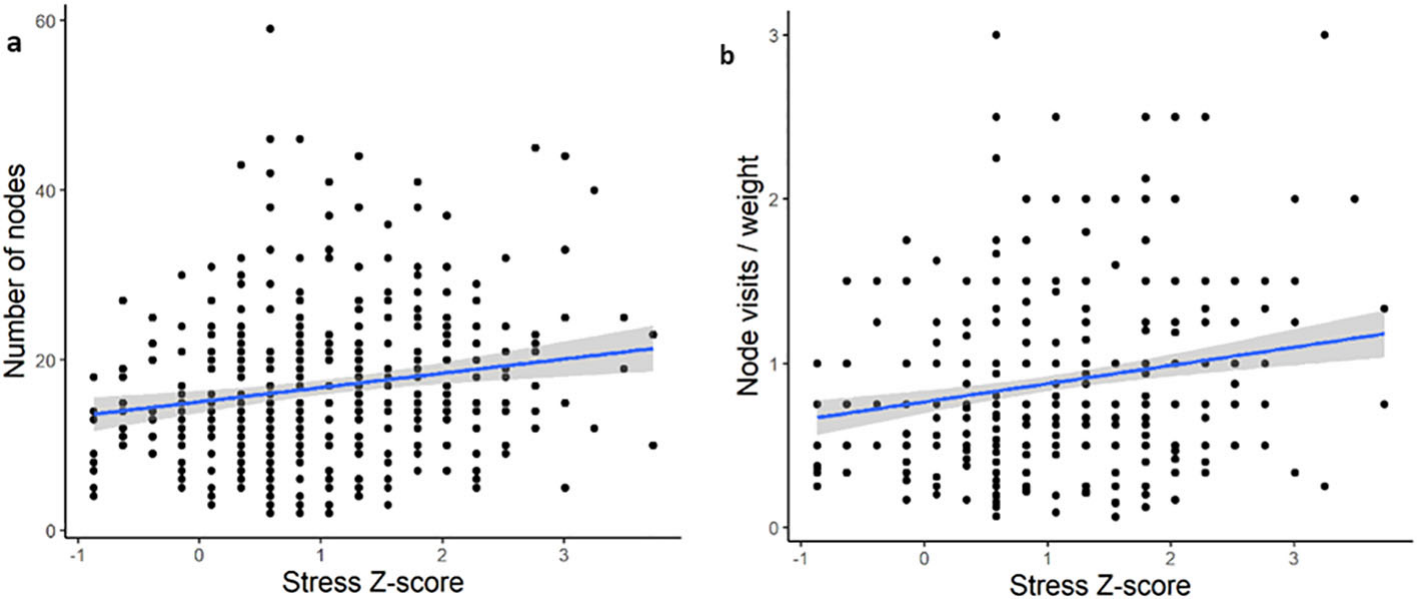
Examples of the linear nature of the relationship between passive sensing features and output (stress) for the best performing features. **(A)** Individual feature of number of unique nodes (r = 0.17). **(B)** Engineered feature of combined average node visits per day with 75^th^ quantile of graph weight (r = 0.21).

We first performed inferential statistics determine which features, if any, show significant relationship with the output (**Figure 2**). Although twenty features significantly correlated with stress z-score, the correlation coefficients (-0.15-0.17) indicated a weak relationship between the features and output. To further confirm our initial finding, we used linear regression with number of unique nodes as a predictor and the stress z-score as the value. Similarly, although the overall fit was statistically significant (*p* < 0.0001) it demonstrated a very low R^2^ value (R^2^ = 0.04, F=7.56) and the data points were scattered, indicating that the model is unable to explain the variance in stress z-score. We then tried to perform feature engineering, including weights of graphs, whereby we combined existing features together to examine the correlation with output. Although this slightly improved the correlations, they were still weak (-0.19-0.21). We further confirmed this by developing and training a ridge linear model on a training dataset and testing the model on a held-out dataset. This resulted in an RMSE of 0.87, suggesting that the model was misclassifying by an entire category of users.

Our initial findings highlighted that weak correlations don’t result in predictive power. One way to improve the performance of our predictive models is to discretize the output variable into a few groups. This would shift away from a regression-type problem towards classification.

### 3.3 Multi-class classification is similarly unable to predict stress

**Figure 3:**
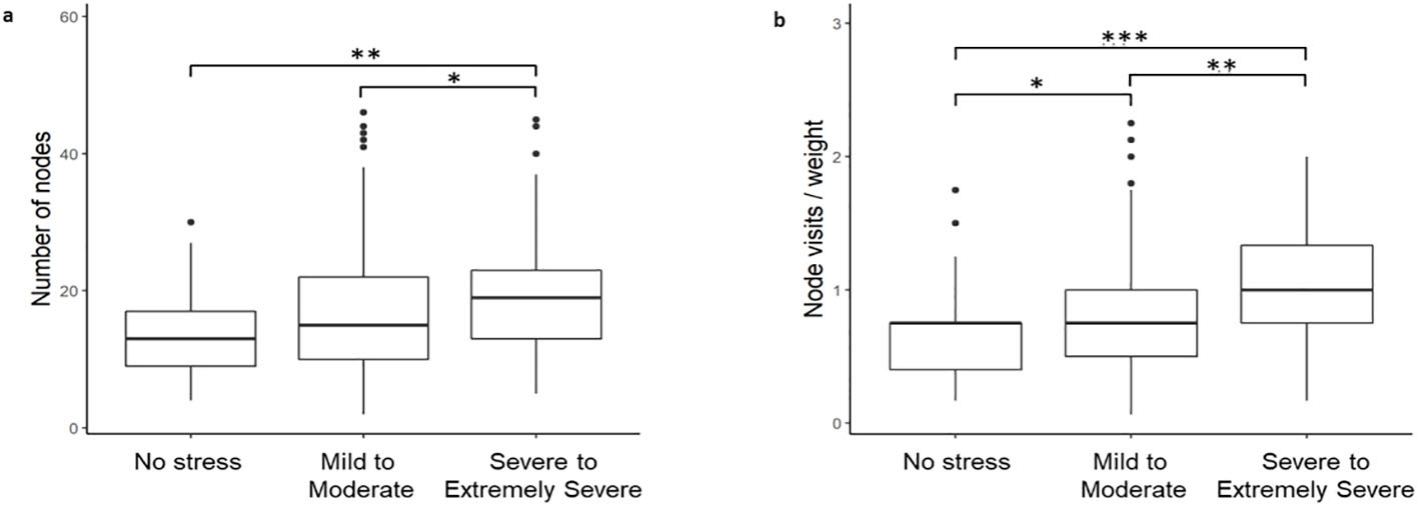
Stress z-scores binned into three categories: no stress (<0.5), mild to moderate (0.5-2), and severe to extremely severe (>2). **(a)** Per individual feature of number of unique nodes. **(b)** Per engineered feature of combined average node visits per day with 75th quantile of graph weight.

We binned the stress z-scores into three categories: no stress (<0.5), mild to moderate (0.5 to 2), and severe to extremely severe (>2). To determine if the use of stress as a continuous, rather than categorical, variable was affecting the ability to develop predictive models, we next binned the stress z-scores into three categories: no stress (<0.5), mild to moderate (0.5 to 2), and severe to extremely severe (>2). A Kruskal-Wallis (χ^2^ = 11.03, df = 2, *p* = 0.004) with *post-hoc* Dunn test confirmed that there was a statistically significant differences in the number of unique nodes between these groups (**Figure 3**).

After successfully binning the stress z-scores into three distinct groups, we next developed a multi-class classification model, CART, to identify if any of the features were now able to predict stress. The model, however, demonstrated very low predictive power (AUC <0.5).

### 3.4 The mild-moderate stress group impacts predictive power in a binary classification

We next sought to identify the potential source of our models’ low predictive power. One possibility was the inclusion of the mild to moderate stress group. The rationale for this was twofold. First, there is evidence that mild to moderate stress can be beneficial, including improving performance and efficiency on dual tasks (37) and concentration (38). It may be the case, therefore, that while some university students may find stress overwhelming others may benefit from mild to moderate stress. This possibility, therefore, suggests that the mild to moderate group is likely heterogenous and highly variable. Evidence for this can also be seen in **Figure 2** whereby the variability in number of nodes and node visits per weight is higher in the mild to moderate users (z-score 0.5 to 2). There is also substantial overlap between the mild to moderate group with the no stress and severe to extremely severe groups on these two passive sensing measures (**Figures 3A, B**). An additional consideration was more severe cases of stress in university students are likely to co-occur with clinical mental health diagnoses, including major depression (39). Therefore, we wanted to assess whether we could improve the clinical translatability by identifying whether digital phenotyping via passive sensing data could differentiate the not stressed from the severely stressed.

After removing the mild to moderate group, we re-performed a binary classification to see if our passive sensing features could predict whether a user had no or severe to extremely severe stress. Using inferential statistics, we first demonstrated that removal of the mild to moderate group improved both the correlation coefficient and significance of the relationship between features and stress (**Table 3**).

**Table 3:** Examples of features correlated with the stress z score in original dataset and trimmed dataset with no mild to moderate stress group.

Features	Original Dataset with Three Groups		No Mild to Moderate Group	
	Correlation Coefficient	*p* value	Correlation Coefficient	*p* value
Number of unique nodes	0.17	0.002	0.37	0.0001
Number of nodes visited per day (75^th^ quantile)	0.14	0.003	0.36	0.0002
Average number of steps per day	-0.14	0.003	-0.25	0.002

Given that we were able to substantially improve the correlation coefficient, we then deployed several predictive machine learning models to determine if these correlations were sufficiently strong enough to be good classifiers. Using the AUC metric, our models showed satisfactory performance (**Table 4**). Despite this, however, the precision and/or recall for all but one models was low, indicating that the models struggled to predict at least one of the two groups. We then developed and deployed a neural network to help overcome this and were indeed able to improve the performance metrics, suggesting that it was able to successfully distinguish between users who were not stressed and those who were severely to extremely severely stressed.

**Table 4:** Machine learning algorithm performance metrics for predicting users with no stress vs. severe to extremely severe stress.

Machine Learning Algorithm	Precision	Recall	F1	AUC
Classification and regression trees (CART)	0.17	0.50	0.26	0.68
Random Forest	0.24	1	0.38	0.67
Shrinkage discriminant analysis (SDA)	0.47	0.67	0.55	0.69
General linear model	0.24	0.80	0.36	0.67
K nearest neighbor	0.41	0.70	0.52	0.70
Neural network	0.82	0.78	0.80	0.79

### 3.5 Further polarizing the no stress and severe to extremely severe stress groups continues to improve predictive power of binary classification models

Although it was clear that the mild to moderate group was indeed affecting the predictive power of our models, only our neural network (out of 6 different supervised machine learning models) performed to a sufficiently high level. This suggested that our findings may be limited with respect to generalizability. To address this, we further increased the minimum z-score of the severe to extremely severe stress group (increased z-score of >2 to z-score of >2.2 to remove those sitting on the “cusp” of severe at 2.1). Again, the rationale for this was that we wanted to determine if there was a potential for clinical translatability of our model to digitally phenotype those who are not stressed relative to those who are experiencing such severe stress that they are high risk of comorbid clinical mental health diagnoses like major depression.

We first confirmed that increasing the minimum z-score of the severe to extremely severe group to 2.2 had a positive effect on the correlation coefficient and statistical significance between our features and groups (**Table 5**).

**Table 5:** Examples of features correlated with the stress z score in original dataset, trimmed dataset with no mild to moderate stress group, and a dataset where severe to extremely severe stress >2.2.

Features	Original Dataset with Three Groups		No Mild to Moderate Group		No Mild to Moderate Group, Severe to Extremely Severe Stress >2.2	
	Correlation Coefficient	*p* value	Correlation Coefficient	*p* value	Correlation Coefficient	*p* value
Number of unique nodes	0.17	0.002	0.37	0.0001	0.41	0.0001
Number of nodes visited per day (75^th^ quantile)	0.14	0.003	0.36	0.0002	0.42	0.0001
Average number of steps per day	-0.14	0.003	-0.25	0.02	-0.27	0.01

We then examined how redefining the severe to extremely severe stress group affected predictive performance in our machine learning models. This improved the models’ performance across all models used and performance metrics. Importantly, redefining the severe to extremely severe stress group improved both the precision and recall, suggesting that our models were indeed able to differentiate between users with no stress and those who were severely to extremely severely stressed (**Table 6**).

**Table 6:** Machine learning algorithm performance metrics for predicting users with no stress vs. severe to extremely severe stress.

Machine Learning Algorithm	Precision	Recall	F1	AUC
Classification and regression trees (CART)	0.5	0.67	0.55	0.55
Random Forest	0.67	0.67	0.67	0.62
Shrinkage discriminant analysis (SDA)	0.67	0.67	0.67	0.74
General linear model	0.75	0.69	0.72	0.68
K nearest neighbor	0.67	0.62	0.64	0.64
Neural network	0.93	0.87	0.83	0.90

**Figure 4:**
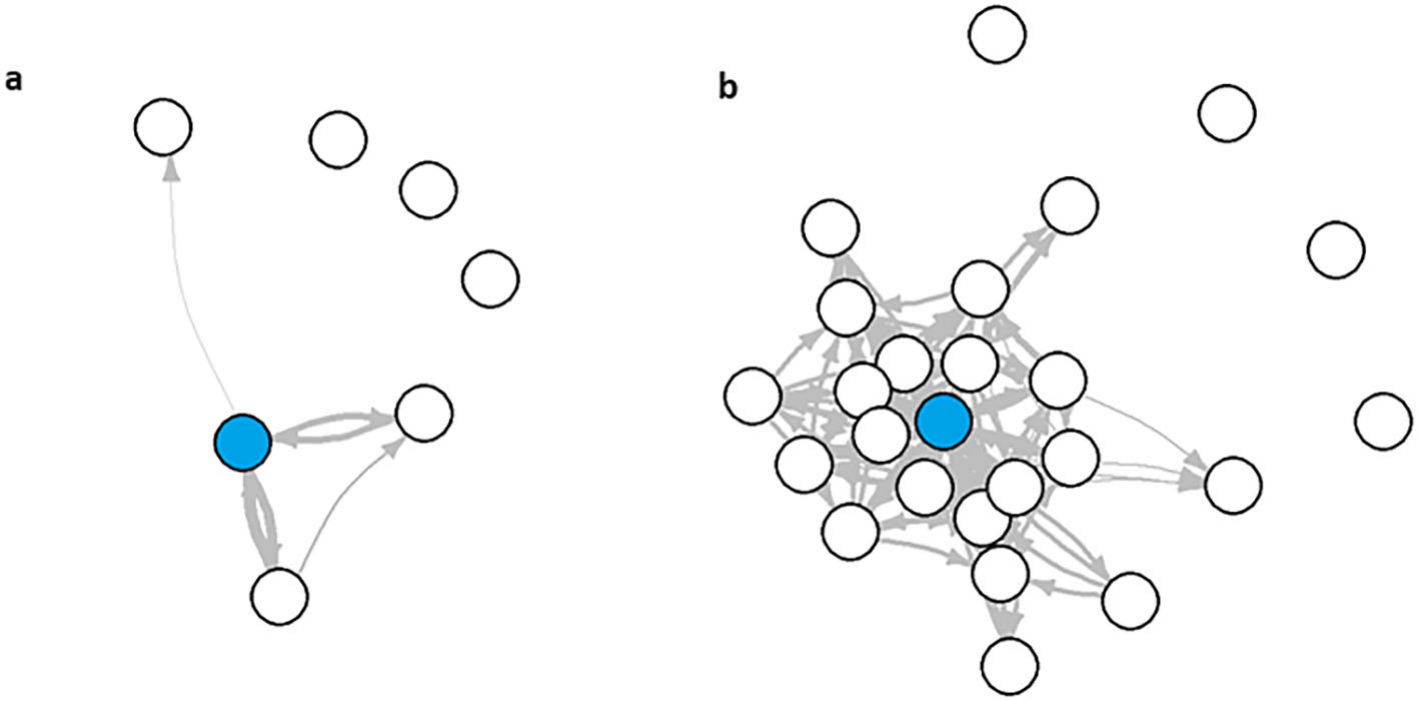
Multigraphs of representative user from the **(A)** no stress and **(B)** severe to extremely severe stress groups.

To further visualize the differences that were predictive of stress status, we built multigraphs of passive sensing features for representative single users from both the no stress and severe to extremely severe stress groups. For the user with no stress, they visited only seven places an average time of once per day across the duration of the study. They also had a 75^th^ quantile path weight of 8.25 (**Figure 4**). The severe to extremely severe user, however, visited 25 places an average of three times per day throughout the study and a 75^th^ quantile path weight of 4 (**Figure 4**).

## 4 Discussion

Using a novel methodological pipeline, we showed that key features from passive sensing data served as a predictor of severe to extremely severe stress across several supervised machine learning models. Key features included number of unique nodes (locations), number of nodes visited per day (75^th^ quantile), and average number of steps per day. These passive sensing features alone were able to differentiate someone who was not stressed versus someone who was severely to extremely severely stressed.

Although two previous studies demonstrated that there was a correlation between GPS features and stress levels in university students, they focused on other features including longer distance between locations (40), evenly distributed time spent at different locations (40), and total distance travelled daily (22). To our knowledge, this is the first paper that shows that a high number of locations, number of locations visited per day, number of steps could predict university students who were severely to extremely severely stressed. Further, this is the first study that has used supervised machine learning to demonstrate that these features can indeed predict the level of stress in university students. This is an important finding in the context of both diagnosis and treatment. First, it suggests that apps that collect passive sensing data may be used to diagnose or predict the level of stress someone is experiencing, allowing us to move away from cumbersome, and at times biased, self-report questionnaires to assess stress (41). Second, our finding suggests that we can use passive sensing data to determine a mental health intervention that may be best suited to a particular user. Personalized mental health interventions have gained popularity with the recent advent of just-in-time adaptive intervention (JITAI) apps. These apps are designed to tailor interventions to the particular needs of the user based on their response to screening questionnaires (e.g. psychological self-reports) (42). Importantly, passive sensing data analyses do not require any additional efforts on the part of the user, highlighting that apps can rapidly tailor or adjust interventions on both immediate and ongoing bases. Future research would benefit from examining whether the inclusion of the passive sensing data features can help to better tailor mental health intervention programs to the individual user.

As discussed, although there are several examples of previous work examining the relationship between GPS data and stress, these works have been limited to correlational analyses and have not fully described the data-specific methodologies. Critically, our study is the first to describe a clear methodological pipeline for extracting features from real-world passive sensing data and, using supervised machine learning, confirm that they can be used for digital phenotyping. With the growing accessibility of passive sensing data sourced from healthcare smartphone apps, there is an urgent need to establish clear methodologies that can be used and replicated by other research groups. First, this marks an important step away from black box style analyses, toward those that are both robust and reproducible. Second, clear methodological pipelines for rapid digital phenotyping from passive sensing data are essential for the success of personalized healthcare apps like JITAIs. Given that we’ve established that digital phenotyping severe to extremely severe stress is possible, there are two next steps for future research. First, our approach should be validated in a cohort of patients with severe stress-related clinical diagnoses such as major depression to establish its clinical translatability and validity. Second, future research should incorporate our methodological pipeline into a JITAI app to establish if it can improve its potential for targeted, personalized interventions.

While this is a promising first step toward using passive sensing data for digital phenotyping, there are some limitations. First, our passive sensing data was unable to digitally phenotype users with mild to moderate stress, as demonstrated by the need to further polarize the Z-scores to the upper limit of 2.2. As discussed, our goal was to test the potential for clinical translatability despite the lack of a clinically diagnosed cohort. More specifically, users with extremely severe levels of stress are at a higher risk of comorbid mental health diagnoses (9, 10). On the other hand, the need to further polarize participants’ stress may be indicative of a high level of heterogeneity in the moderate stress group. Future research, therefore, should focus on two important aspects. First, what is driving the potential heterogeneity of moderate stress. For example, perhaps these people are better able to cope with stress relative to their extremely stressed counterparts. Another consideration is whether some users benefit from their stress levels. Future research, therefore, would benefit from focusing on if there are ways to differentiate between these types of “stressed” users. This could be done, for example, by the inclusion of additional questionnaires to assess users’ subjective experience of their current performance on tasks and ability to handle stress. From an early intervention approach, it is essential to elucidate whether digital phenotyping may be able to predict those at risk of transitioning from mild to moderate stress to more severe stress. This remains an important line of enquiry for future research. Another consideration of the present work is that the users of the Vibe Up app were more likely to be female than male, resulting in a 3:1 ratio of females to males. Although this is in line with previous research of university student mental health app users (43), it suggests that future work needs to establish whether this digital phenotyping extends to male students as well. A final limitation is that we lacked clinical diagnostic data for these users and therefore relied on self-report of symptoms. Considering this, future work should replicate our study and pipeline in a clinical cohort to identify its generalizability to clinical populations.

## 5 Conclusion

In conclusion, we have shown in the present study that passive sensing data from a smartphone application, including number of locations, number of locations visited per day, and average number of steps per day were able to be used to differentiate between a university student who was not stressed versus severely to extremely severely stressed. This work has important implications for further tailoring personalized mental health interventions, including JITAIs, based on data that does not require additional efforts on the part of the user. Despite this, we did find that moderately stressed participants were unable to be digitally phenotyped from the same passive sensing data variables. The reason(s) for this remain unclear but may be due to heterogeneity in this group stemming from variables including, for example, ability to cope with stress and perceiving stress as positive rather than negative. Future work should focus on identifying the sources of this heterogeneity and examining whether our pipeline is generalizable to a cohort with confirmed clinical diagnoses.

## Data Availability

The raw data supporting the conclusions of this article will be made available on request, subject to the relevant governance procedures.
